# Influence of Measuring Circuit Parameters on the Characteristics of MIS-Capacitor Hydrogen Sensors

**DOI:** 10.3390/s26134209

**Published:** 2026-07-03

**Authors:** Nikolay Samotaev, Boris Podlepetsky, Maya Etrekova, Konstantin Oblov

**Affiliations:** Micro- and Nanoelectronics Department, National Research Nuclear University MEPhI (Moscow Engineering Physics Institute), 115409 Moscow, Russia; bipodlepetskij@mephi.ru (B.P.); moetrekova@mephi.ru (M.E.); kyoblov@mephi.ru (K.O.)

**Keywords:** capacitance-voltage (*CV*) characteristic, electricalmodes, MISC gas sensors, thin films

## Abstract

Using electrophysical models of MIS-capacitor gas-sensing elements, the influence of measuring circuit parameters on the metrological characteristics of hydrogen sensors was investigated. Recommendations for selecting optimal measurement circuit modes are provided, both in general and using sensor elements with a Pd-Ta_2_O_5_-SiO_2_-nSi structure as an example. This article presents the results of an analysis and comparative study of three methods for measuring the capacitance of MISC sensors: (a) the AC bridge with a balance indicator (ACB + BI), (b) the divider method (DM), and (c) the bridge method (BM). The advantages and disadvantages of each method for practical implementation in gas analytical instruments are discussed. Furthermore, experimental data on the long-term stability of MISC sensor characteristics are provided, including the sensor response to hydrogen and the zero-point drift.

## 1. Introduction

Hydrogen sensors are widely employed in devices and systems designed for monitoring environmental conditions and ensuring explosion safety across various industrial and civil facilities [1,2]. The development of microelectronic sensors and gas analysis systems based on micro- and nano-technologies is regarded as a promising approach for creating compact means of measuring gas concentrations [3]. A critical requirement for such devices is the technological, thermal, and electromagnetic compatibility of their constituent elements, including sensors as well as primary and secondary transducers. Among solid-state gas-sensitive transducers, capacitors and field-effect transistors with a metal-insulator-semiconductor structure (MISC and MISFET) exhibit excellent compatibility with integrated circuit elements [4].

Gas concentration sensors based on MIS-structures have been extensively studied by numerous research groups [5,6,7]. A significant contribution to the development of such sensors has been made by researchers from Linköping University [8,9,10]. Objectively, this sensor type has not yet gained widespread adoption, as its operational range is primarily limited to low H_2_ concentrations, from hundreds of ppb to 1–2% vol. This range is currently in lower demand compared to the explosive range. Nevertheless, the tasks of early diagnostics for critical events involving trace hydrogen are becoming increasingly relevant, including breath analysis [11,12], early detection of technical equipment malfunctions [13,14] and fire precursors [15,16,17], as well as mineral exploration [18,19], etc. The main advantages of the proposed sensor type are the long-term stability of the sensitive elements’ characteristics and the high reproducibility of their fabrication process, which is compatible with integrated circuit (IC) technology. The development of artificial intelligence (AI) and the resolution of the selectivity problem may ultimately facilitate the broader adoption and practical application of such sensors [20,21,22].

At the National Research Nuclear University MEPhI, sensor elements based on MISC and MISFET with Pd and Pt gates have been developed. These sensors are designed for the detection of hydrogen-containing gases (H_2_ [23], NH_3_ [24], H_2_S [25]) and are based on multilayer structures such as SiO_2_-Si, Si_3_N_4_-SiO_2_-Si, and Ta_2_O_5_-SiO_2_-Si [3,25,26]. Experimental studies have demonstrated that hydrogen sensors utilizing a sensitive element with a Pd-Ta_2_O_5_-SiO_2_-Si structure on a silicon MOS crystal exhibit superior performance characteristic. Furthermore, the influence of various factors on the metrological and operational properties of these sensors has been investigated. It has been shown that these characteristics are dependent on design and technological parameters, chip temperature, operating time, and environmental conditions [23,24,27,28].

The aim of this study is to evaluate the influence of the electrical operating modes of measurement circuits on the performance characteristics of MISC-based hydrogen sensors. The investigation is conducted in a general context and illustrated by a specific example of a sensor utilizing a Pd-Ta_2_O_5_-SiO_2_-nSi structure. For the interpretation of the obtained experimental data, updated physical models and equivalent circuit diagrams were employed. These models take into account the influence of various external and internal factors. It is anticipated that the proposed models and gas concentration measurement techniques will serve as a foundation for predicting the performance of sensors with more complex circuit topologies, including those incorporating one or multiple MISC elements.

## 2. Initial Data and Performance Models of MISC Hydrogen Sensors

**Figure 1:**
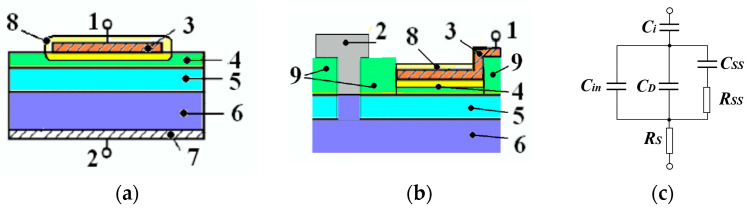
Vertical (**a**) and planar (**b**) schematic diagrams of MISC structures: 1 and 2 terminals of working electrode 3 and ohmic contact 7 (**a**) or region 6 (**b**); 4 and 9 thin and thick dielectric films; 5 and 6 high-resistance and low-resistance regions of n- or p-type semiconductor; 8 gas-sensitive active regions. (**c**) Equivalent electrical circuit.

Hydrogen sensors can be implemented with either a vertical or planar structure of the sensing element. Simplified schematic diagrams illustrating the structure of the MISC sensing element in the cross-section view of the device and its equivalent electrical circuit are presented in Figure 1. The maximum sensitivity of sensors based on MISC structures is observed at elevated temperatures of active region 8 (380–500 K) (see Figure 1a,b). The heating and stabilization of the operating temperature are provided by integrated heating and temperature-sensitive elements within the sensor design [24]. Furthermore, it should be noted that the electronic circuits employed for measuring gas concentration and maintaining temperature stability have a significant impact on the metrological characteristics of the sensor [29].

In the equivalent circuit shown in Figure 1c, the capacitors correspond to the following capacitances:

- *C_i_* of the thin dielectric film;
- *C_in_* of the inversion layer;
- *C_D_* of the space-charge region (depletion layer);
- *C_ss_* effective capacitance of the surface states at the dielectric–semiconductor interface.

The resistors *R_s_* and *R_ss_* represent the total resistance of semiconductor regions 5 and 6 and the resistance that determines the switching time (*τ_ss_* = *C_ss_R_ss_*) of the surface-state charges with density *Q_ss_*, respectively.

**Figure 2:**
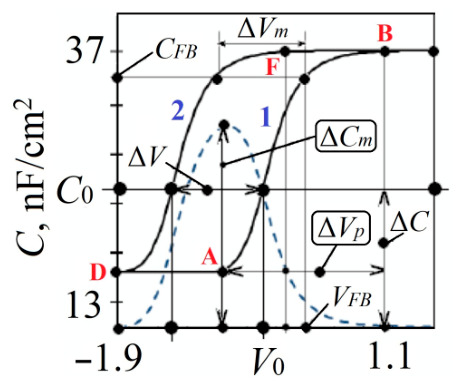
Examples of high-frequency *CV* characteristics of the MISC structure are shown before (curve 1) and after (curve 2) exposure to hydrogen. The dashed line illustrates the capacitance change, Δ*C*, resulting from a *CV* characteristic shift of Δ*V* within the voltage range Δ*V_m_*. The maximum capacitance change, Δ*C_m_*, is indicated for the operating voltage *V_A_*.

Studies have shown that the general operating principle of hydrogen-sensitive MISC sensors is independent of their structural type and manufacturing technology. Hydrogen exposure induces physicochemical reactions at the various interfaces of the working electrode: at the gas/metal and metal/dielectric boundaries, as well as within the bulk of the metal and dielectric. These processes depend on the gas concentration *N* and lead to changes in the work function difference potential *φ_ms_* and the effective charge density in the dielectric, *Q_te_*. As a result, the capacitance-voltage (*CV*) characteristic (the dependence of capacitance *C* on voltage *V*) shifts along the voltage (*V*) axis parallel to its original position, as illustrated in Figure 2.

We examined the quantitative changes in the *CV* parameters of MISCs based on n-type silicon as a function of various factors. Subsequently, for the operating voltage *V* = *V*_12_ < 0, the capacitance *C* can be represented, in the general case, as a function of the surface potential *φ_s_* using Equations (1) and (2):

*V*(*φ_s_*) = *φ_ms_* − [*Q_te_* + *Q_ss_*(*φ_s_*) + *Q_s_*(*φ_s_*)]/*C_i_* + *φ_s_* = *V*_0_(*φ_s_*) − Δ*V*(*N*) (1)

*C*(*φ_s_*) = *C_i_*[*C_in_*(*φ_s_*) + *C_D_*(*φ_s_*) + *C_ss_*(*φ_s_*)]/[*C_i_* + *C_in_*(*φ_s_*) + *C_D_*(*φ_s_*) + *C_ss_*(*φ_s_*)] (2)

where, in the inversion region (*φ_s_* > *φ_s_*_0_), the effective charge density *Q*_s_(*φ*_s_) in n-Si is calculated as

Q_s_(φ_s_) = *Q_d_*(*φ_s_*) + *Q_in_*(*φ_s_*) ≈ *a*·*C_i_*{*φ_s_* + *φ_T_* exp[(*φ_s_* − 2*φ_s_*_0_)/*φ_T_*]}^1/2^

**Figure 3:**
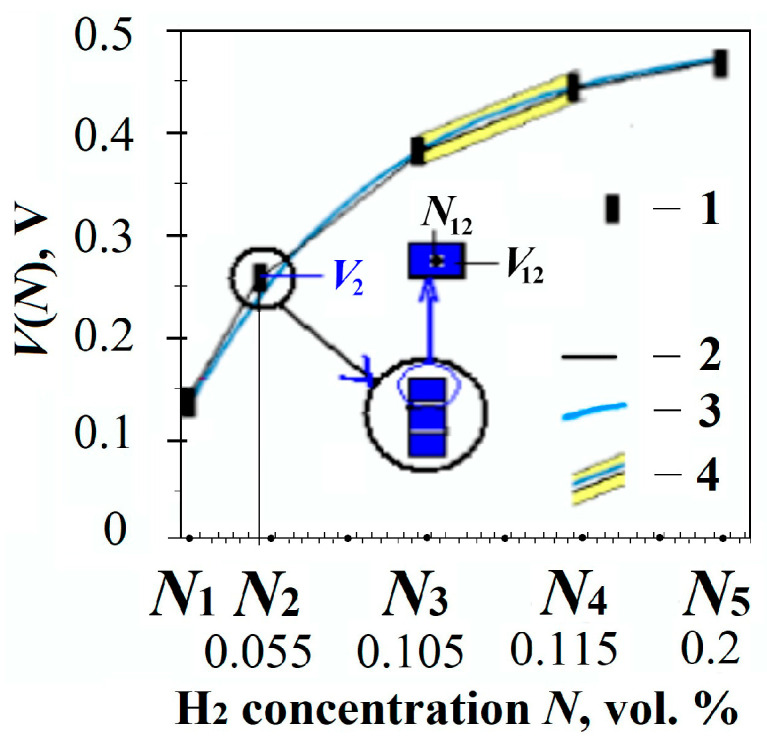
The formation of the experimental dependence *V*(*N*) (data points 1 and connecting lines 2) and the approximated dependence (curve 3), along with a fragment of the error band (4). The number 1 indicates the range of amplitudes for *m* = 5 responses, corresponding to their *n* = 3 measurements.

The transfer function models *V*(*N*) are defined as approximations of the averaged experimental dependences *V_ki_*(*N_ki_*), based on the data of *m* complete *i*-th responses, each measured *n* times, where *k* = 1, 2, …, *n* and *i* = 1, 2, …, *m* [28]. Typically, to determine the transfer function for a specific sensor series, *m* complete responses are obtained from a sample of *n* sensor units (here, indices *k* and *i* denote the ordinal numbers of the sensor samples and responses, respectively). If the absolute errors of the measuring instruments are Δ*N_ki_* and Δ*V_ki_*, then the true values of each (*ki*)-th measurement are located on the *V*(*N*) graph within a rectangular field with sides 2Δ*N_ki_* and 2Δ*V_ki_*, centered at the point (*N_ki_*, *V_ki_*), as shown in Figure 3.

The sensitivity of the *CV* characteristic to hydrogen and external factors {*z_j_*} acting on the MISC (background gases, temperature, radiation) is manifested provided that the overlap coefficient *K_C_* = *C_B_*/*C_A_* > 1 (Figure 2) and the slope *g_C_* = *d_C_*/*d_V_* exceeds the ratio of the measurement errors, i.e., *g_C_* > Δ*C_C_*/Δ*V_V_*, where Δ*C_C_* and Δ*V_V_* are the absolute errors of capacitance and voltage measurement, respectively. The dependence *V*(*N*, *C_i_*) determines the hydrogen sensitivity of the MISC, which is quantified by the voltage sensitivity coefficient *S_V_* = *d_V_*/*d_N_*. To mitigate the influence of the stray capacitances *C_ss_* and *C_in_* in MISC sensors, the operating region is selected based on high-frequency *CV* characteristics (*f* > 50 kHz) in the depletion and weak inversion modes, where the surface potential is constrained to the interval |*φ_s_*| ∈ (0: 2*φ_s_*_0_]. Consequently, the dependencies |*V_0_*(*φ_s_*)|, Δ*V*(*N*) and *C*(*φ_s_*) are represented as

|*V*_0_(*φ_s_*)| *= φ_s_* + *a*{*φ_s_* + *φ_T_*·exp[(*φ_s_* − 2*φ_s_*_0_)/*φ_T_*)]}^1/2^ *+ b*(*φ_s_*_0_ − *φ_s_*) + *φ_ms_*_0_ − *Q_te_*_0_/*C_i_* (3)

Δ*V*(*N*, *C_i_*) = Δ*φ_ms_*(*N*) − Δ*Q_te_*(*N*)/*C_i_* (4)

*C*(*φ_s_*) = *C_i_C_D_*(*φ_s_*)/[*C_i_* + *C_D_*(*φ_s_*)] = *C_i_*/[1 + *F_C_*(*V*)] (5)

*F_C_*(*φ_s_*) = 2{*φ_s_* + *φ_T_*·exp[(*φ_s_* − 2*φ_s_*_0_)/*φ_T_*]}^1/2^/{*a* + *a*·exp[(*φ_s_* − 2*φ_s_*_0_)/*φ_T_*]} (6)

where *φ_s_* ≡ |*φ_s_*|, *C_i_* = *C_max_* = *ε*_0_*ε*/*d*, *a* = [2*ε*_0_*ε_s_qN_D_*]^1/2^/*C_i_* and *b* = *qN_ss_*/(*φ_bg_C_i_*) are the design and technological parameters, generally designated as {*p_k_*}. Electrophysical models (3)–(6) were used for the quantitative analysis of the MISC sensors’ characteristics with the Pd-Ta_2_O_5_-SiO_2_-*n*Si structure, the parameters of which are presented in Table 1. Accurate modeling of the *CV* characteristics is possible only by numerical methods. By specifying the values of the surface potential φ*_s_*, one can calculate *V*(φ*_s_*) using Equation (3), determine the corresponding values of the inverse function *φ_s_*(*V*), and, by substituting these into Equations (5) and (6), obtain the dependences *C*[*φ_s_*(*V*)]. The potential *φ_ms_*_0_, the capacitance *C_i_*, and the dependence *F_C_*(*φ_s_*) can be calculated theoretically, whereas the dependence Δ*V*(*N*) and the charges *Q_te_*_0_ and *Q_ss_*_0_ are determined experimentally.

The electrophysical models of hydrogen sensitivity proposed in the first part of the article in general form can serve as a basis for the development of “more complex designs” (for example, sensors with several MISC elements) and can be integrated into computer-aided design systems. For example, a sub-circuit schematic of model implementation using a VCVS (voltage-controlled voltage source φ_s_) that connects in series with the gate VG of the standard MIS-structure done in a Verilog-A-model [30].

## 3. Results

In addition to the conversion function *V*(*N*) and the differential sensitivity *S_V_* = *dV/dN*, the main metrological characteristics of the MISC include the following:

- Sensitivity: *S_i_* = Δ*V_i_/*Δ*N_i_*
- Sensitivity Threshold: *N*_0_ = Δ*V_V_/S_Vm_*
- Measurement Range: Δ*N_m_* ∈ [*N*_0_; *N_m_*]
- Absolute Error Δ(*N*) and Relative Error *δN* = 100% × Δ(*N*)/*N*
- Operating Conversion Range: Δ*N*_12_ ∈ [*N*_1_; *N*_2_] for a given maximum error *δN_m_*
- Response Speed (Bandwidth): determined by the rise (τ_0.9_) and fall (τ_0.1_) times of the voltage *V_i_*(*t*) in response to a concentration pulse *N_i_*

For the studied MISC, in the concentration range from a few ppm to 1 vol.%, the dependencies of the response signal Δ*V*(*N*) and sensitivity *S_V_*(*N*) can be approximated as follows [25]:

Δ*V*(*N*) = Δ*V_m_* [1 − exp(−*k_N_ N*)] and *S_V_* = *k_N_* Δ*V_m_* exp(−*k_N_ N*) (7)

where Δ*V_m_* = Δ*Q_tem_*/*C_i_* − Δ*φ_msm_*. The values of the parameters Δ*V_m_*, Δ*Q_tem_*, *C_i_*, Δ*φ_msm_*, and *k_N_* were determined experimentally to be (0.5 ± 0.02) V, (14.4 ± 0.5) nC/cm^2^, (37 ± 1) nF/cm^2^, −(0.11 ± 0.005) V, and (8.0 ± 0.3) (%)^−1^, respectively.

*N*(Δ*V*) = (1/*k_N_*) ln[Δ*V_m_*/(Δ*V_m_* − Δ*V*)] = 0.125 ln[1/(1 − 2Δ*V*)] (8)

In electrophysical models (1)–(6), capacitance and charge values are represented as specific values (per unit area), which does not affect the qualitative analysis of the *CV* characteristics. Models (3)–(8), parameters {*p_k_*}, and capacitance/voltage measurement errors are key quantities for the quantitative assessment of the influence of circuit electrical modes on the metrological characteristics of MISCs.

**Figure 4:**
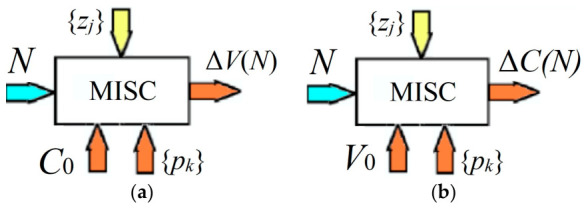
Two options for determining the concentration *N* based on the shift of the *CV* characteristic: (**a**) measuring the voltage shift Δ*V* at a constant capacitance (using scheme 1); (**b**) measuring the capacitance shift Δ*C* at a constant voltage (using scheme 2).

Two approaches can be used to determine the *N* concentration based on the leftward shift of the *CV* characteristic by Δ*V*(*N*) (Figure 2, Table 2):

1. Measure Δ*V* at a constant capacitance *C*_0_ ∈ [*C_min_* + Δ*C_C_*; *C_max_* − Δ*C_C_*] using scheme 1 (Figure 4a);
2. Measure Δ*C* at a constant voltage *V*_0_ ∈ [*V*_A_;*V*_B_) (Figure 2) using scheme 2 (Figure 4b).

At *T* = 400 K, the average parameter values are *Q_te_*_0_ ≈ 20 nC/cm^2^, *N_ss_*≈ 10^11^ cm^−2^, *a* = 1.11 V^1/2^ and b = 0.43. According to the data in Figure 3, the real concentration values are expressed as *N*_*i*p_ = *N_i_* ± Δ*N_i_*. Assuming that the instrumental error Δ*N_ki_* is constant for all samples and equal to Δ*N_N_*, the absolute error Δ*N_i_* is determined as

Δ*N_i_* = Δ*N_N_* + Δ*V_i_*/│*S_i_*│ (9)

Of practical interest is the relative error

δ*N*(*N*) = 100%·{[Δ*N_N_*·│*S_d_*│ + Δ(*V*)]/(*N*·│*S_d_*│)} (10)

where Δ(*V*) denotes the total absolute error, which may be a function of the parameter *N*. This value comprises the instrumental error Δ*V_V_*, errors induced by external influencing factors Δ*V_Z_*, and the spread of circuit parameters Δ*V_ep_*. The minimum attainable value of Δ(*V*) is defined by the Δ*V_V_*.

According to Equation (7), the intrinsic differential sensitivity to hydrogen, *S_V_*, is a function of the parameters Δ*Q_tem_*, *C_i_*, Δ*φ_msm_* and *k_N_*. These parameters are, in turn, determined by the initial characteristics of the MISC, namely the sets {*z_j_*} and {*p_k_*}. Notably, the value of *S_V_* is independent of the electrical operating modes of the measuring circuit.

In circuit 1, an increase in the parameter *N* at a fixed value of *C*_0_ causes the operating point (*C*_0_, *V*_0_) to shift leftward. This shift occurs from the initial range │*V*_0_│ ∈ (*V_A_*; *V_B_*) by a magnitude Δ*V* ∈ (0 V; 0.5 V) (see Figure 2). For instance, at *C*_0_ = 26 nF/cm^2^, the operating voltage is │*V*_0_│ = 0.4 V. Furthermore, an increase in *C*_0_ leads to a decrease in │*V*_0_│ and a corresponding increase in the relative sensitivity of the circuit. The sensitivity *S*_1_ lies within the following range: *S*_1_ ∈ [3*e*^−8*N*^, 20*e*^−8*N*^] vol^−1^%. The instrumental error of the circuit, Δ(*V*), is either constant or, in the presence of a relative voltage measurement error *δV*, exhibits a dependence on *N* according to the following expression:

Δ(*V*) = 0.01·*δV*{*V*_0_ + 0.5[1 − exp(−8*N*)]}

**Figure 5:**
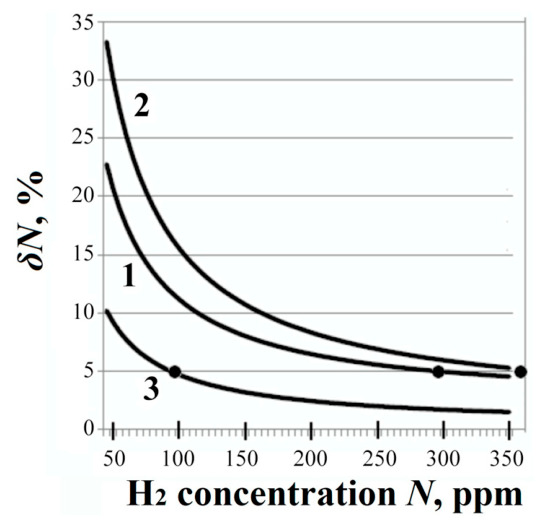
The dependence of the *δN*(*N*) on the average concentration *N*. Curves 1, 2, and 3 correspond to the following error conditions: *δV* = 1%, Δ(*V*) = 5 mV, and Δ(*V*) = 1 mV. The absolute error is Δ*N_N_* = 2 ppm. The dots mark the upper limits of the operating ranges for *δN_m_* = 5%.

**Figure 6:**
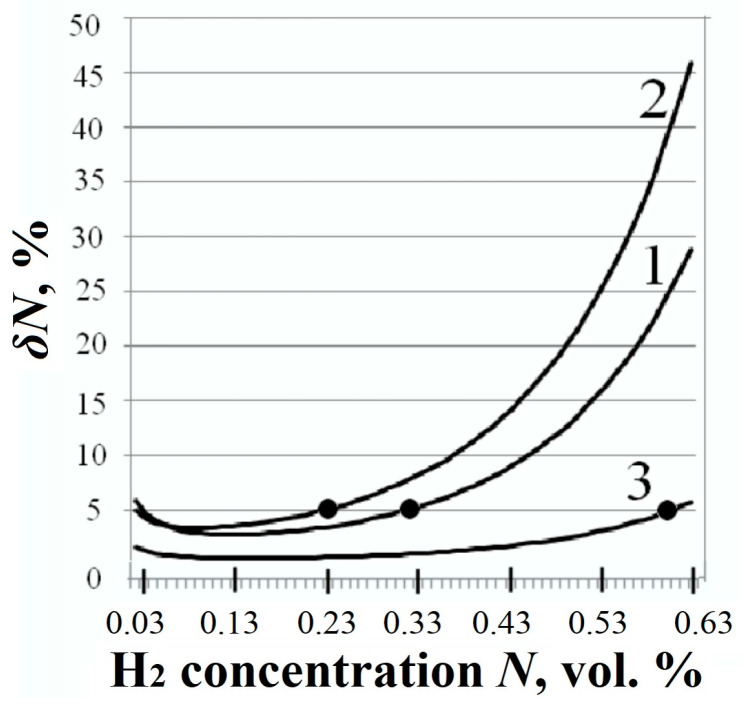
The dependences of the *δN*(*N*) on the high concentration values. For notation, see the caption of Figure 5.

If the instrumental error is Δ*V_V_* = 1 mV, the sensitivity threshold is *N*_0_ = 2.5 ppm. The maximum measurement range is defined as Δ*N_m_* ∈ [2.5 ppm;1.55 vol.%]. For hydrogen in air, the conversion factor is approximately 1 vol.% ≈ 10^4^ ppm. The working conversion range Δ*N*_12_ ∈ [*N*_1_; *N*_2_] for a specified maximum error *δN_m_* is determined from the functional dependence *δN*(*N*), as illustrated in Figure 5 and Figure 6.

In circuit 2, an increase in *N* causes the operating point (*C*_0_, *V*_0_) to shift upward by Δ*C* ∈ (0;Δ*C_m_*) at a constant │*V*_0_│ ∈ (*V_D_*;*V_B_*). The maximum shift Δ*C_m_* is reached at │*V*_0_│= *V_A_* (see Figure 2). The value of Δ*C_m_* depends on the “swing” of the operating region of the *CV* characteristic, defined as Δ*V_p_* = (*V_B_* − *V_A_*) ≈ 2.5 V, and on Δ*V_m_* = 0.5 V. For the MISC under consideration, where Δ*V_m_* < Δ*V_p_*, the maximum value is Δ*C_m_* ≈ 4 nF/cm^2^ at *V_A_* = −1.4 V. Conversely, for sensors with Δ*V_m_* > Δ*V_p_*, the value of Δ*C_m_* = (*C_i_* − *C_min_*) ≈ 22 nF/cm^2^ and is determined by the initial parameters {*p_k_*}.

For circuit 2, the optimal initial coordinates of the operating point are *V*_0_ = −1.4 V and *C*_0_ = *C_min_* + Δ*C_C_* = 15.15 nF/cm^2^.

With an increase in the magnitude of │*V*_0_│, both the capacitance *C*_0_ and the slope *g_C_* decrease, while the relative sensitivity of the *S*_2_ circuit increases, reaching a maximum value of *S*_2max_= 0.27*g_C_*·exp(−8*N*) vol^−1^%. This increase does not exceed the relative sensitivity *S*_1_ within the operating range of the *CV* characteristics, as *g_C_* < *C*_0_/*V*_0_ holds true. The error of the measuring circuit, Δ*C_C_*, is either constant or depends on *δC* according to the relation Δ*C_C_* = 0.01δ*C*·*C*_0_. The error Δ(*V*), as defined by Equation (10), is given by Δ(*V*) = Δ*C*/*g_C_* and depends on the parameter *N*. This error influences the conversion limits of the MISC sensor.

**Figure 7:**
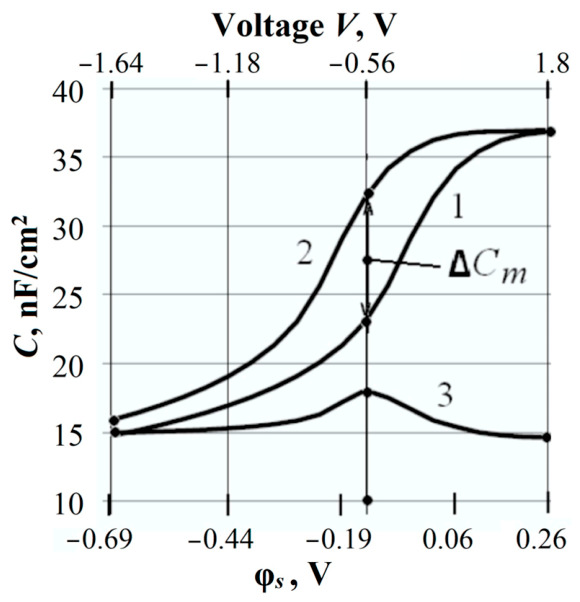
Dependences of *C*(*φ_s_*) before (1) and after (2) exposure to H_2_. Curve (3) represents *C*(*φ_s_*) = *C_min_* + ∫*S_C_*d*φ_s_.*

Each value of *φ_s_* corresponds to a capacitance difference Δ*C*(*φ_s_*) = *C*_2_(*φ_s_*) − *C*_1_(*φ_s_*), which is equal to the integral ∫*S_C_*d*φ_s_* (see Figure 7). This difference defines the capacitive sensitivity *S_C_* = d(Δ*C*)/d*φ_s_* and the slope *g_C_* = *S_C_*(d*φ_s_*/d*V*). The dependences *S_C_*(*φ_s_*) and *g_C_*(*φ_s_*) are determined by the initial parameter set {*p_k_*}. The minimum, average, and maximum values of the capacitive sensitivity are *S_C_*_0_ = 3 nF/(V·cm^2^), *S_C_* ≈ 24 nF/(V·cm^2^), and *S_Cm_* ≈ 70 nF/(V·cm^2^), respectively.

Analysis of the obtained data reveals that the metrological characteristics of the specific type of MISC sensor are influenced by its intrinsic hydrogen sensitivity *S_V_*(*N*) and the errors associated with the measurement circuitry. During modeling, the analytical function *S_V_*(*N*) is determined by approximating the averaged experimental dependences Δ*V(N)*, while taking into account the uncertainties of the model parameters.

The boundaries of the MISC conversion range depend on the following:

- The chosen approach to determining the *N* concentration based on the Δ*V*(*N*) shifts in the *CV* characteristic;
- The initial coordinates of the operating point (*C*_0_, *V*_0_);
- The instrumental errors of the measuring circuits and the error of Δ(*V*).

**Figure 8:**
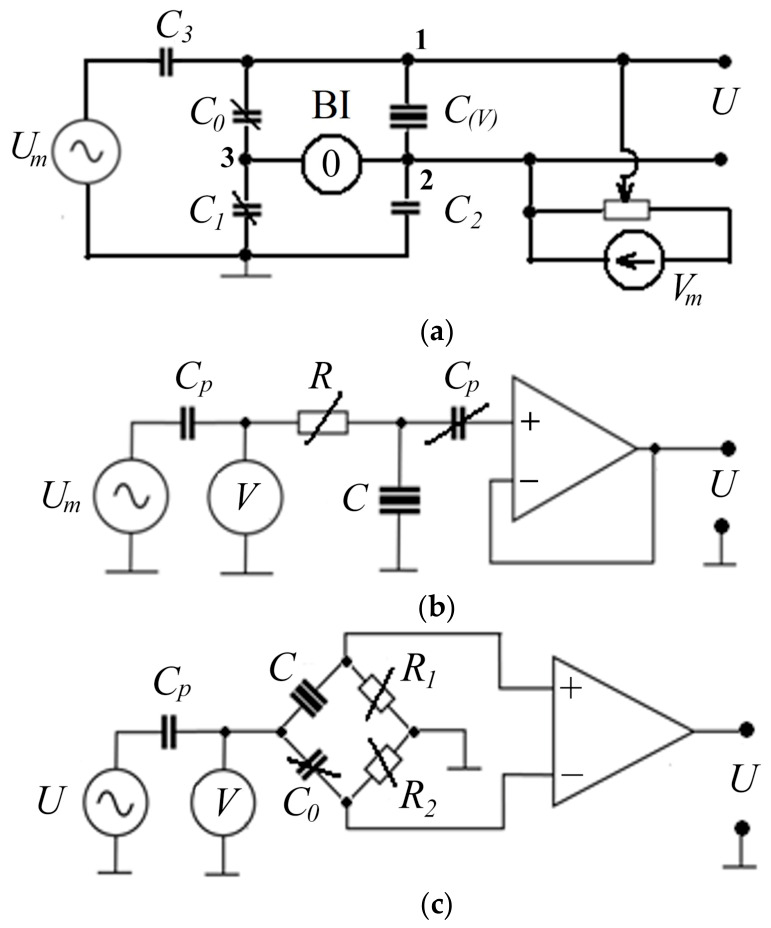
Circuit diagrams: (**a**) AC bridge with balance indicator (ACB + BI),AC/DC sources (*U_m_*, *V_m_*), MISC capacitor *C*(*V*), and calibration capacitors (*C*_0_, *C*_1_); (**b**) divider method (DM); (**c**) bridge method (BM).

In practice, experimental or production-scale devices with specified metrological characteristics are employed to measure capacitance and *CV* characteristics. These instruments operate on various physical principles (Figure 8) [31]. For preliminary studies of MISC sensors under development, when their characteristics are still unknown, AC bridge circuits equipped with a balance indicator (ACB + BI) are utilized, as illustrated in Figure 8a. The wide Δ*V* measurement range is the main advantage of this circuit. However, the complexity of the circuit structure and calibration, as well as the stringent requirements for the ND offset adjustment, limit its applicability in practical hydrogen sensors.

Common methods for measuring capacitance include the divider method (DM) and the bridge method (BM) (Figure 8b,c). In all cases, the measured capacitance values are not specific (i.e., normalized) but absolute values, which depend on the working electrode area (for the MISC under study, *C_i_* = 370 pF).

A circuit based on the DM is recommended for measuring gas concentrations at the maximum allowable concentration (*MAC*) level in residential and occupational areas. While a BM circuit provides the maximum sensitivity for the MISC sensor, it inherently limits the measurable concentration range. It should be noted that an increase in the range of measurable capacitances and concentrations leads to a reduction in sensitivity. In this regard, the DM occupies an intermediate position, offering a balanced trade-off between sensitivity and the measurement range.

The boundary values of the conversion ranges of the MISC hydrogen sensor for various circuit configurations and error margins are exemplified in Table 3.

The selection of circuit design, the method for evaluating hydrogen sensitivity, and the optimal electrical conditions indirectly influence the metrological and operational characteristics of MISC hydrogen sensors. This influence is mediated by instrumental and statistical errors inherent in the measurement of *CV* characteristics. Consequently, a reduction in these errors leads to an expansion of the sensor’s conversion range.

## 4. Experimental Validation

Tests of electronic boards, fabricated using various capacitance measurement techniques, were conducted with the same MISC hydrogen sensor operating at a temperature of 100 °C (370 K). The relevant settings and characteristics of the boards are presented in Table 4. The experimental MISC sensor was fabricated using a well-established technology, which is described in greater detail in our previous publications [32].

**Figure 9:**
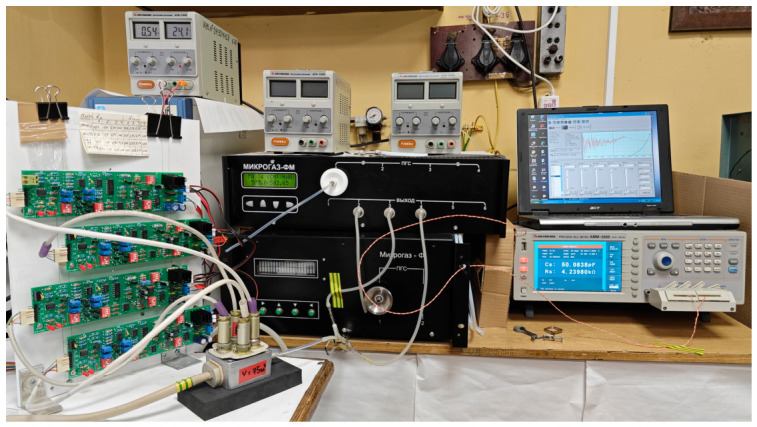
Experimental setup for evaluating the hydrogen sensitivity of MISC sensors.

To investigate hydrogen sensitivity, a dynamic gas mixing setup was employed, utilizing cylinders containing air and a test gas mixture of air + H_2_ (0.1 vol%). After mixing, the resulting gas mixture was supplied to the chamber of the MISC sensor, which had a volume not exceeding 0.1 L. A photograph of the experimental setup is presented in Figure 9. Cylinders with hydrogen (JSC MGPZ, Moscow, Russia) were diluted with clean air using a Mikrogaz generator-diluent (JSC Intera, Kirovo-Chepetsk, Russia)

**Figure 10:**
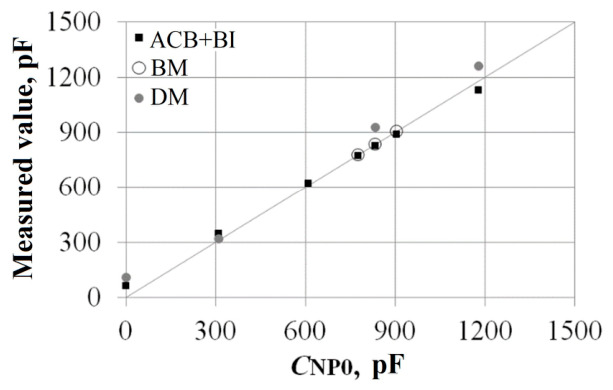
Verification of capacitance measurement accuracy on electronic boards.

The accuracy of setting and maintaining the V_0_ values on the boards was no worse than ±0.1 mV. For the operating temperature of the MISC sensor, the accuracy was no worse than ±0.1 °C. Additionally, prior to testing with the MISC sensor, the accuracy of capacitance measurements performed by the electronic boards was verified. For this purpose, temperature-stable NP0 ceramic capacitors (±30 ppm/°C) were used, and their capacitance was monitored with an AMM-1130 multimeter (Aktakom Ltd., Moscow, Russia) (±1%). The results are presented in Figure 10.

As shown in Table 4, the capacitance measurement range of the BM circuit is significantly narrower compared to the ACB + BI and DM circuits and is determined by the reference capacitance *C*_0_ (see Figure 2). Consequently, while the BM circuit enables high-accuracy measurement of the MISC sensor capacitance, this is achievable only within a limited range of Δ*C* = 130 pF, which substantially restricts its practical applicability. It is worth noting (see Figure 10) that the measurement error for the ACB + BI and DM circuit boards also depends on the measurement range. The highest accuracy for the DM circuit board is attained when calibrating the operating capacitance within a range not exceeding 1500 pF; beyond this limit, the error increases by 8% for every additional 1000 pF.

**Figure 11:**
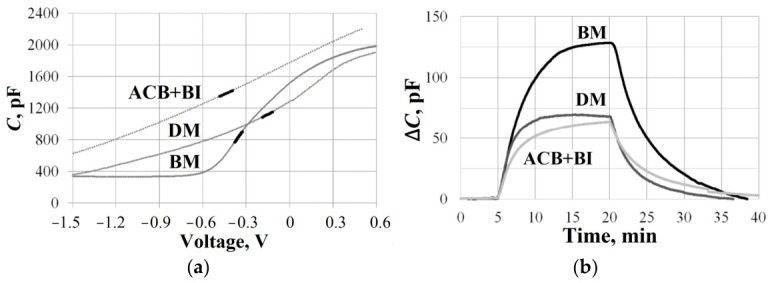
Analysis of the output signals of the MISC sensor using different capacitance measurement methods: (**a**) *CV* characteristics, with the section corresponding to the shift under 5 ppm hydrogen highlighted in bold; (**b**) dynamic response to 5 ppm hydrogen.

The *CV* characteristics of the control MISC sensor, measured using the electronic boards, are presented in Figure 11a. Note that the sensor’s CV characteristics were measured in clean air and under 5 ppm hydrogen over the entire bias voltage range. The sections are highlighted in bold for clarity to avoid cluttering the graph while clearly indicating the position of the operating point (*V*_0_) and the magnitude of the gas-induced shift (Δ*V*).

Due to differences in the measurement signal parameters implemented in the electronic boards, the shape of the *CV* curve for the same sensor varies depending on the measurement scheme. The operating points for each case were selected according to the method described above (see Figure 2), after which the dynamic characteristics of the MISC sensor were investigated in response to a hydrogen concentration of 5 ppm. The results are represented in Figure 11b and Table 5. Note that the greater capacitive response of the sensor, observed during measurements on the electronic board using the BM, is attributed to the steeper slope of its *CV* characteristic (∆*C*/∆*V*). Consequently, the magnitude of the capacitance change (∆*C* = 128 pF) under 5 ppm H_2_ is twice as large as that obtained with the other two methods, while exhibiting a significantly smaller shift in the bias voltage (∆*V* = −50 mV).

**Figure 12:**
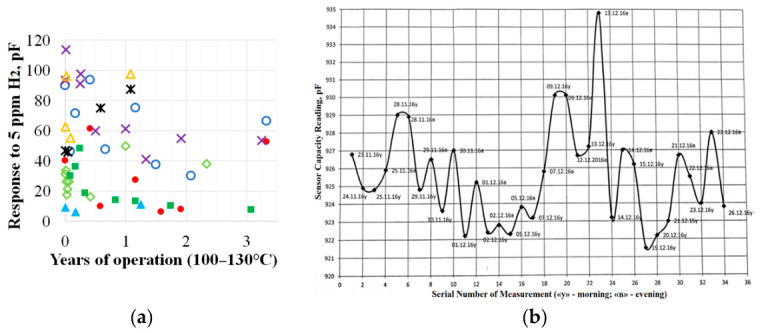
(**a**) Long-term stability of MISC sensors response to 5 ppm H_2_ at 100 °C. Different symbols in the figure body correspond to the dynamics of sensor sensitivity (relative to the initial level) for various MISC sensor samples fabricated using PLD technology but differing in dielectric material during long-term operation in ambient air for approximately 8–10 hours a day, 5 days a week. (**b**) Zero-reading drift over a 1-month observation period.

Influence of measuring circuit parameters on the characteristics of MIS-capacitor hydrogen sensors also must be compared with the accuracy of the sensors’ response to hydrogen concentration during its lifetime, particularly noting the gas sensor characteristics that are of practical importance for implementation. These include long-term stability of characteristics, as well as the influence of interfering factors, such as humidity and background gases, to which the sensors may exhibit cross-sensitivity. Experimental data on the long-term stability of sensitivity, obtained using the BM capacitance measurement method, are presented in Figure 12.

The experiment involved the continuous operation of a batch of eight hydrogen sensors for over three years at a temperature of 100–130 °C, with periodic verification of their operating parameters, specifically hydrogen sensitivity. Hydrogen measurements were conducted in static mode. A 0.5% vol. H_2_ Standard Gas Mixture was injected into a sealed 1 L container filled with room air using a 1 cm^3^ medical syringe. This resulted in a test H_2_ concentration of 5 ppm. As shown in Figure 12a, the sensitivity of the majority of sensors exhibited a slight decrease relative to the initial level, typically stabilizing within the first six months of operation. Observations of the zero-reading drift (Figure 12b) revealed periodic fluctuations on a monthly scale, likely attributable to gradual changes in environmental parameters. As can be seen, the fluctuations in sensor readings did not exceed 10–20 pF, which, according to the sensor’s preliminary calibration, is equivalent to a nominal change in ambient hydrogen concentration of no more than ±1 ppm.

The issues of humidity influence, its compensation algorithms, and cross-sensitivity have been studied in detail and described in our previous work [32,33].

## 5. Conclusions

This paper presents an analysis of the methods and tools for evaluating the metrological performance of MISC sensor devices. This study is based on electrophysical models of the electrical characteristics of metal-insulator-semiconductor capacitive sensors. As a case study, this paper examines hydrogen sensors with a Pd-Ta_2_O_5_-SiO_2_-*n*Si structure. Furthermore, recommendations are provided for the selection of optimal circuit configurations to determine the operating characteristics of sensors for detecting hydrogen-containing gas concentrations.

The bridge capacitance measurement circuit has been demonstrated to be well-suited for laboratory applications where high accuracy and sensitivity are of paramount importance. For the determination of gas concentrations at the MAC level in residential and occupational environments, a circuit based on the divider method is recommended. While the bridge circuit provides maximum sensitivity for the MISC sensor, it inherently limits the measurable range of gas concentrations. An increase in the range of measurable capacitances and corresponding concentrations results in a reduction in sensitivity. The divider method offers a compromise, occupying an intermediate position in terms of both sensitivity and measurement range.

The proposed models and methodology for studying the characteristics of MISC-based hydrogen sensors can serve as a foundation for the mathematical support required in the design of gas analyzers utilizing various types of MISCs. This includes the development of more complex designs and the prediction of their performance. Furthermore, engineering-physical models, parameterized with specific numerical values for given MISC structures, can be integrated into computer-aided design (CAD) systems for integrated gas concentration sensors.

## Figures and Tables

**Table 1:** Parameters of the MISC with a Pd-Ta_2_O_5_-SiO_2_-*n*Si structure and the models used.

Designations	Parameters	Average Values
*ε*_1_, *ε*_2_ and *ε_s_*	relative permittivity of Ta_2_O_5_, SiO_2_ and Si	25, 4 and 12
*N* *_D_*	donor concentration in Si	5 × 10^15^ cm^−3^
*d*_1_ and *d*_2_	thickness of Ta_2_O_5_ and SiO_2_	90 nm and 80 nm
ε_0_	relative permittivity of vacuum	8.85 × 10^−12^ F/m
*k*	Boltzmann constant	1.38 × 10^−23^ J/K
*q*	electron charge	1.6 × 10^−19^ C
*d*	thickness of thin dielectric (*d*_1_ + *d*_2_)	170 nm
*s_e_*	effective area of the working electrode	0.01 cm^2^
ε	effective permittivity (*d*ε_1_ε_2_)/(ε_1_*d*_2_ + ε_2_*d*_1_)	7.2
*C* *_i_*	specific capacitance of a thin dielectric (ε_0_ε)/*d*	37 nF/cm^2^
*a*	charge parameter in Si [(2*q*·*ε*_0_∙*ε_s_*∙*N_D_*)^1/2^*/C_i_*]	1.1 V^1/2^
*b*	charge parameter *Q_ss_* at *N_ss_* = 10^12^ cm^−2^ [*qN_ss_*/(*φ_bg_C_i_*)]	0.43
*φ_ms_*	work function difference potential between Pd and Si	φ*_ms_*_0_ = 85 mV
*T*	MISC chip temperature	400 K
*φ_T_*	thermal potential (*kT/q*) at 400 K	33 mV
*φ_gb_*	band gap potential in Si	1.08 V
*φ_s0_*	donor level potential [*φ_T_*ln(*N_D_/n_i_*)]	0.34 V
*φ_s_*	surface potential [*φ*(SiO_2_-Si) − *φ_F_*]	(0.05…0.8) V
*Q_te_* and *Q_ss_*	charge density in the dielectric and at the SiO_2_-Si interface	(2…100) nC/cm^2^
*N_ss_*	density of surface states at the boundary SiO_2_-Si	(10^11^…10^13^) cm^−2^

**Table 2:** Approaches to determining the concentration *N* based on the shifts of the *CV* characteristic.

Scheme #	Control Parameters	Output Values	Relative Sensitivities
1	*C*_0_ ∈ (*C_min_* + Δ(*C*); *C_max_* − Δ(*C*))	Δ*V*(*N*) = Δ*V_m_* [1 − exp(−*k_N_ N*)]	*S*_1_* = k_N_*Δ*V_m_* exp(−*k_N_ N*)/*V*_0_
2	*V*_0_ ∈ [*V*_A_; *V*_B_)	Δ*C*(*N*) *= C*(*V*_0_ − Δ*V*(*N*)) *− C*(*V*_0_)	*S*_2_* = g_C_ k_N_*Δ*V_m_* × exp(−*k_N_ N*)/*C*_0_

**Table 3:** Boundaries of the MISC sensor conversion range for various measurement errors.

Δ(*V*)	*N*_0_, ppm	*N*_1_, ppm	*N*_2_, ppm	*N_m_*, ppm	δ*N*_min_, %
0.01·│*V*(*N*)│	9	295	2300	7400	3.17
5 mV	140	355	3150	8100	2.72
1 mV	4.5	95	6000	10,450	0.54
0.6 mV (Δ*C_C_* = 1 pF)	2.5	65	8200	13,300	0.43

**Table 4:** Configuration parameters and performance characteristics of the electronic boards.

Comparison Parameters	Capacitance Measurement Method		
	ACB + BI	DM	BM
*F*, kHz	8	20	18
*A*, mV	1500	1000	200
intrinsic noise of the electronic board *, ±pF	0.05	0.1	0.13
noise of the readout circuitry *C_noise_*, ±pF	0.15	0.2	0.26
measurement range, pF (±δ, %)	C_0_ ± 50 (±1) 400…1200 (±5) 300…5000 (±11)	500…2000 (±1) 300…5000 (±30)	C_0_ ± 65 (±1)

* Values obtained using NP0 capacitors.

**Table 5:** MISC sensor hydrogen sensitivity data.

Comparison Parameters	Capacitance Measurement Method		
	ACB + BI	DM	BM
operating point coordinate *V*_0_, mV	−500	−200	−400
*CV* characteristic shift of Δ*V*, mV	−74	−70	−50
capacitance change ∆*C*, pF	64	70	128
hydrogen sensitivity *S*_C_(*N*), pF/ppm	12.8	14	25.6
response speed *τ*_0.9_; *τ*_0.1_; *τ_full_*, min	9; 14; 25	6; 9; 20	8; 11; 30
calculated value of the detection limit *N_LOD_H2_*, ppb	35	43	30.5

Designations: *τ_full_*—characteristic time for the sensor readings to return to their original values after removal of hydrogen exposure; *N_LOD_H2_* = (3∙*C*_noise_)/*S*_C_ (see Table 4).

## Data Availability

The original contributions presented in this study are included in the article. Further inquiries can be directed to the corresponding author.
